# Benzimidazole-Based Schiff Base Hybrid Scaffolds: A Promising Approach to Develop Multi-Target Drugs for Alzheimer’s Disease

**DOI:** 10.3390/ph16091278

**Published:** 2023-09-11

**Authors:** Rafaqat Hussain, Shoaib Khan, Hayat Ullah, Farhan Ali, Yousaf Khan, Asma Sardar, Rashid Iqbal, Farid S. Ataya, Nasser M. El-Sabbagh, Gaber El-Saber Batiha

**Affiliations:** 1 Department of Chemistry, Hazara University, Mansehra 21120, Pakistan; rafaqathussain0347@gmail.com (R.H.); asmasardar@gmail.com (A.S.); 2 Department of Chemistry, Abbottabad University of Science and Technology (AUST), Abbottabad 22020, Pakistan; 3 Department of Chemistry, University of Okara, Okara 56130, Pakistan; 4 Department of Chemistry, COMSATS University, Islamabad 45550, Pakistan; 5 Department of Agroecology-Climate and Water, Aarhus University, Blichers Allé 20, 8830 Tjele, Denmark; rashid.iqbal@iub.edu.pk; 6 Department of Agronomy, Faculty of Agriculture and Environment, The Islamia University of Bahawalpur, Bahawalpur 63100, Pakistan; 7 Department of Biochemistry, College of Science, King Saud University, P.O. Box 2455, Riyadh 11451, Saudi Arabia; fataya@ksu.edu.sa; 8 Department of Veterinary Pharmacology, Faculty of Veterinary Medicine, Alexandria University, Alexandria 21526, Egypt; nasserelsabbagh@yahoo.com; 9 Department of Pharmacology and Therapeutics, Faculty of Veterinary Medicine, Damanhour University, Damanhour 22511, AlBeheira, Egypt; dr_gaber_batiha@vetmed.dmu.edu.eg

**Keywords:** synthesis, benzimidazole, Schiff base, SAR, AChE, BuChE and molecular docking

## Abstract

A series of benzimidazole-based Schiff base derivatives (**1**–**18**) were synthesized and structurally elucidated through ^1^H NMR, ^13^C NMR and HREI-MS analysis. Subsequently, these synthetic derivatives were subjected to evaluation for their inhibitory capabilities against acetylcholinesterase (AChE) and butyrylcholinesterase (BuChE). All these derivatives showed significant inhibition against AChE with an IC_50_ value in the range of 123.9 ± 10.20 to 342.60 ± 10.60 µM and BuChE in the range of 131.30 ± 9.70 to 375.80 ± 12.80 µM in comparison with standard Donepezil, which has IC_50_ values of 243.76 ± 5.70 µM (AChE) and 276.60 ± 6.50 µM (BuChE), respectively. Compounds **3**, **5** and **9** exhibited potent inhibition against both AChE and BuChE. Molecular docking studies were used to validate and establish the structure–activity relationship of the synthesized derivatives.

## 1. Introduction

**Figure 1:**
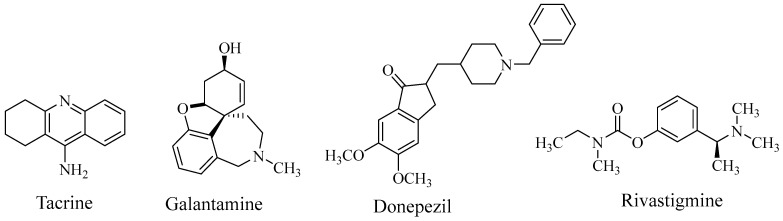
Clinically approved drugs for Alzheimer’s disease.

Alzheimer’s dementia (AD) is a well-known neurodegenerative condition affecting the aging population [1,2,3]. AD is characterized by a gradual progression within the brain, initiating years prior to the manifestation of visible symptoms [4]. Early clinical indications of AD also include memory issues with names, events, or conversations from recent interactions. Some of the later symptoms include decreased communication, disorientation, confusion, strange behavior, poor judgment, swallowing, walking difficulties, and deterioration in speech [5]. While a comprehensive understanding of the underlying causes and origins of the disease is still in progress, the histopathological alterations observed in the brains of patients encompass the presence of extracellular amyloid β-protein (Aβ) accumulation within amyloid plaques [6], as well as the formation of intraneuronal neurofibrillary tangles [7]. AD is not currently managed in any way. The treatments for AD that are currently available only offer symptomatic alleviation; they cannot alter the course of the disease [8]. Aβ and τ-protein development, which lowers levels of brain acetylcholine (ACh), is related to increased acetylcholinesterase (AChE) enzyme activity and the loss of muscarinergic neurons [9]. Numerous drugs intended for Alzheimer’s disease patients have been medically approved, such as Donepezil and galantamine. These drugs are designed to selectively target AChE. In contrast, compounds like rivastigmine and tacrine have been identified as BuChE and AChE inhibitors (Figure 1) [10].

**Figure 2:**
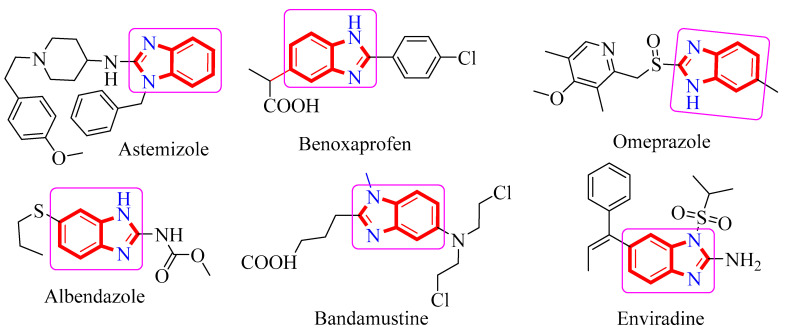
Benzimidazole motif bearing bioactive drugs.

It has been demonstrated that benzimidazole and its analogs have a variety of biological profiles, including those that act with antihypertensive [11,12], anti-Alzheimer [13], antimicrobial, antiviral [14], anti-diabetic [15,16], and anticancer characteristics [17]. Some bioactive drugs with intriguing biological profiles, such as astemizole, albendazole, bendamastin, candesartan, enviradine, omeprazole, and benzimidazole, are useful in medicinal chemistry due to their heterocyclic nature (Figure 2) [18].

**Figure 3:**
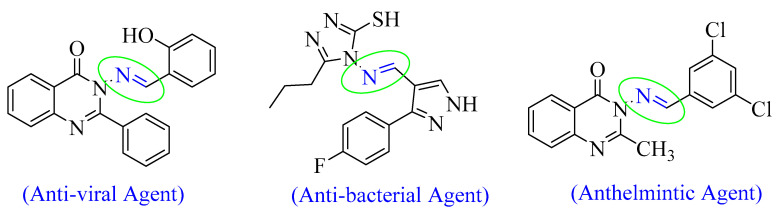
Bioactive drugs with a Schiff base skeleton in their core structure.

Schiff base and its scaffolds have been reported to have numerous significant biological profiles due to their interesting biological activities. In addition, heterocyclic scaffolds with the Schiff base moiety were known to demonstrate a broad spectrum of therapeutic and biological potentials, including anti-helminthic [19], antimicrobial [20], antitumor [21], and antiviral agents (Figure 3) [22].

**Figure 4:**
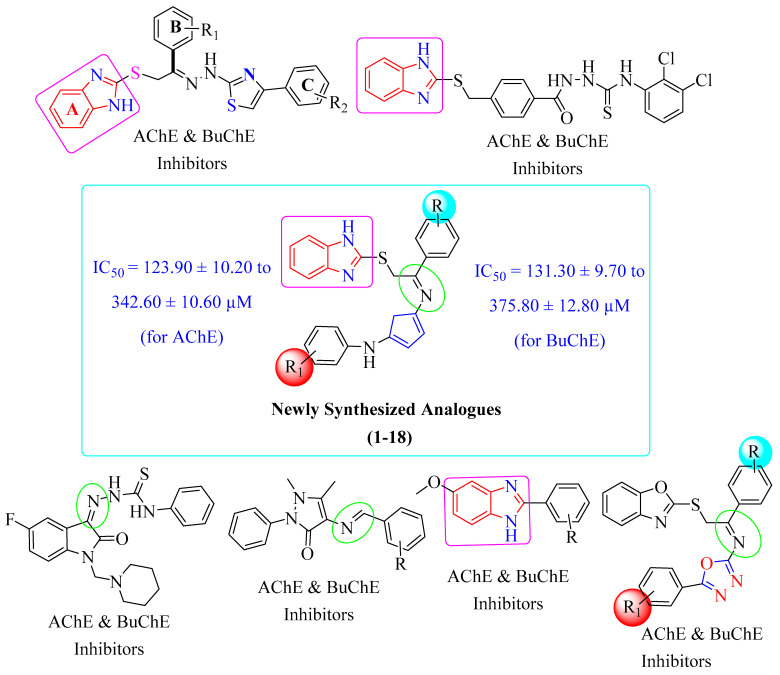
Rationale study of the present work.

Keeping in mind the biological significance of benzimidazole [23,24,25] and Schiff base [26,27,28] derivatives, in this study, we synthesized benzimidazole-based Schiff base hybrid scaffolds to further explore the AChE and BuChE inhibition in search of lead molecules (Figure 4).

## 2. Results and Discussion

### 2.1. Chemistry

The synthesis of benzimidazole-based Schiff base compounds (**1**–**18**) was carried out through a multi-step procedure. In the initial step, carbon disulfide was gradually mixed with a solution of benzene-1,2-diamine (I) and stirred in a mixture of ethanol and water (1:1). Following this, KOH was introduced, and the resulting solution was refluxed for a period of 5 h. This led to the formation of 2-mercaptobenzimidazole, serving as substrate (**II**). Then, the intermediate (**II**) underwent further reflux with phenacyl bromides in the presence of ethanol and triethylamine for approximately 8 h. Subsequently, the surplus solvent was evaporated, yielding the formation of S-substituted benzimidazole, identified as intermediate (**III**).

**Scheme 1:**
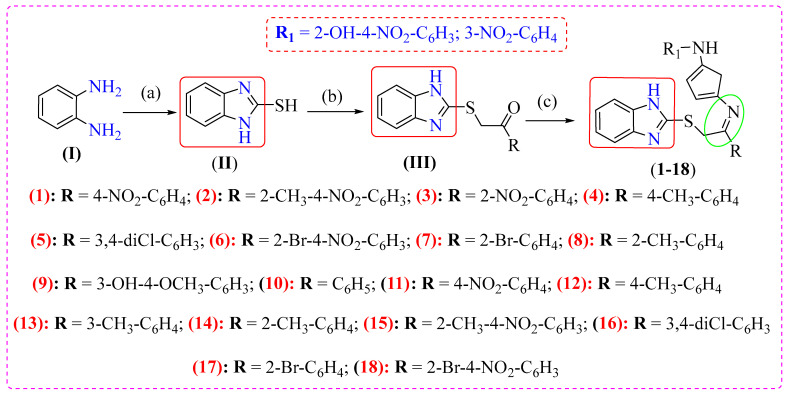
A synthetic pathway to produce benzimidazole-based Schiff base derivatives; (**a**) CS_2_, KOH, EtOH:H_2_O, reflux 5 h; (**b**) phenacyl bromide, EtOH, Et_3_N, Reflux 8 h; (**c**) *N*^1^-phenylcyclopenta-3,5-diene-1,3-diamine, Acetic acid (glacial), Reflux 6 h.

In the final stage, substrate (**III**) underwent condensation by being stirred with substituted N1-phenylcyclopenta-3,5-diene-1,3-diamine in glacial acetic acid. The resultant residue was continuously stirred and confirmed through a reflux process lasting 6 h. Ultimately, this series of reactions successfully yielded the desired benzimidazole-based Schiff base (**1**–**18**) (Scheme 1).

### 2.2. Acetylcholinesterase and Butyrylcholinesterase Inhibition Studies

The in vitro enzymatic activities of benzimidazole-based Schiff base derivatives (**1**–**18**) were examined to assess their impact on acetylcholinesterase (AChE) and butyrylcholinesterase (BuChE). Results are shown in Table 1. Both AChE and BuChE enzymes were actively inhibited by the complete range of benzimidazole-based Schiff base derivatives (IC_50_ values = 123.90 ± 10.20–342.60 ± 10.60 µM against AChE and 131.30 ± 9.70 to 375.80 ± 12.80 µM against BuChE). Donepezil was employed as a standard inhibitor, and its IC_50_ values were determined to be 243.76 ± 5.70 µM for AChE and 276.60 ± 6.50 µM for BuChE. Compounds **3**, **5**, and **9** showed particularly significant activity. Their respective IC_50_ values for AChE were 123.9 ± 10.20, 168.60 ± 7.70, and 174.22 ± 6.76 µM, while those for BuChE were 131.30 ± 9.70, 179.30 ± 6.70, and 181.20 ± 8.50 µM, respectively. Furthermore, the other synthesized Schiff base derivatives also effectively inhibited the targeted enzymes and even showed stronger inhibitory potency than Donepezil.

#### Structure–Activity Relationship (SAR) for Acetylcholinesterase and Butyrylcholinesterase Inhibition Studies

**Figure 5:**
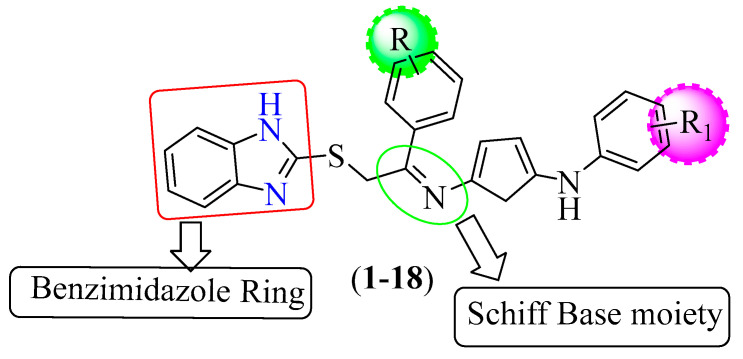
General representation of benzimidazole-based Schiff base analogues (**1**–**18**) for SAR analysis.

To determine and simplify SAR for AChE and BuChE inhibition, the Schiff base derivatives (**1**–**18**) were divided into two groups based on the nature and position of substituents as R and R^1^ groups. Its group comprises compounds **1**–**8**, where R^1^ represents OH at the 2 position and NO_2_ at the 4 position of the phenyl ring (Figure 5).

Amongst compounds **1**–**8**, compound **3** (IC_50_ = 123.90 ± 10.20 µM (AChE), IC_50_ = 131.30 ± 9.70 µM (BuChE), and **5** (IC_50_ = 168.60 ± 7.70 µM (AChE), IC_50_ = 179.30 ± 6.70 µM (BuChE) showed significant inhibition against both AChE and BuChE enzymes. This might be due to the presence of 2-NO_2_ and 3,4-diCl group as R. Compounds **1** (IC_50_ = 213.78 ± 11.60 µM (AChE), IC_50_ = 248.70 ± 10.90 µM (BuChE) bearing 4-NO_2_, and compound **2** (IC_50_ = 198.82 ± 10.20 µM, IC_50_ = 203.70 ± 8.20 µM (BuChE) bearing 2-CH_3_ and the 4-NO_2_ group as R also showed good inhibition against both enzymes. When the NO_2_ group was replaced by the CH_3_ group, a slight decrease in inhibitory activity was observed, as in compounds **4** (IC_50_ = 273.50 ± 7.60 µM (AChE), IC_50_ = 285.30 ± 8.20 µM (BuChE), **8** (IC_50_ = 290.70 ± 12.60 µM (AChE), IC_50_ = 303.60 ± 13.80 µM (BuChE), and **6** (IC_50_ = 226.30 ± 10.10 µM (AChE), IC_50_ = 273.70 ± 13.80 µM (BuChE)), bearing the CH_3_ group at the 4 and 2 positions, while compound **6** was bearing the 2-Br and 4-NO_2_ group, respectively. In compound **7** (IC_50_ = 313.60 ± 12.80 µM (AChE), IC_50_ = 325.80 ± 13.70 µM (BuChE)), bearing the 2-Br group, a decrease in inhibitory activity was observed as compared to other compounds, but these derivatives were more active than the standard drug Donepezil. Now, we switch toward the second group of synthesized derivatives that comprises compounds **9**–**18,** based on the R^1^ group, where R^1^ represents the 3-NO_2_ group at the phenyl ring, as depicted in Table 1.

Amongst the derivatives **9**–**18**, compound **9** (IC_50_ = 174.22 ± 6.76 µM (AChE), IC_50_ = 181.20 ± 8.50 µM (BuChE)) was found to be the most active compound that showed strong inhibition against both enzymes. This compound holds 3-OH and 4-OCH_3_ groups as R. When the 4-OCH_3_ was replaced with 4-NO_2_ as in compound **11** (IC_50_ = 267.50 ± 11.70 µM (AChE), IC_50_ = 273.60 ± 9.10 µM (BuChE)), a marginal decline in inhibitory activity was observed for both enzymes. In compounds **12**–**14** (IC_50_ = 288.90 ± 11.50–320.67± 13.50 µM (AChE), IC_50_ = 310.70 ± 12.30–354.10 ± 12.70 µM (BuChE)), when the CH_3_ group was introduced at the 2, 3, and 4 positions, respectively, the inhibitory activity of these compounds declined for both enzymes. Compound **10** (IC_50_ = 342.60 ± 10.60 µM (AChE), IC_50_ = 375.80 ± 12.80 µM (BuChE)) bearing the diCl group at the 3,4-position also exhibited a high IC_50_ value. A good inhibitory activity was observed for compounds **16** (IC_50_ = 209.45 ± 8.30 µM (AChE), IC_50_ = 218.50 ± 8.50 µM (BuChE)), bearing the diCl group at the 3,4-position, **17** (IC_50_ = 233.82 ± 10.10 µM (AChE), IC_50_ = 269.40 ± 9.60 µM (BuChE)) bearing 2-Br, and **18** (IC_50_ = 213.50 ± 8.20 µM (AChE), IC_50_ = 246.60 ± 9.10 µM (BuChE)) bearing 2-Br and 4-NO_2_ group as the R group (Table 1).

Based on the SAR analysis, it can be inferred that benzimidazole-based Schiff base derivatives carrying 2-OH and 4-NO_2_ substitutions on the phenyl ring (referred to as R^1^) showcased favorable inhibitory potential. However, alterations in the substituents located at the phenyl ring (referred to as R) also played a role in influencing variations in inhibitory activity. To reinforce these findings, a docking study was performed, shedding light on the interactions occurring between the synthesized derivatives and the active sites of the said enzymes.

### 2.3. Molecular Docking

**Figure 6:**
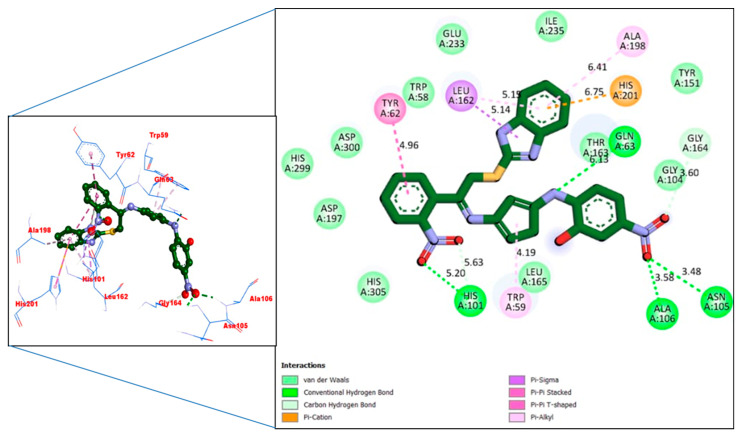
Protein–ligand interaction (PLI) analysis for the most potent compound **3** with the targeted AChE enzyme, along with its 3D (**left**) and 2D (**right**) representations.

**Figure 7:**
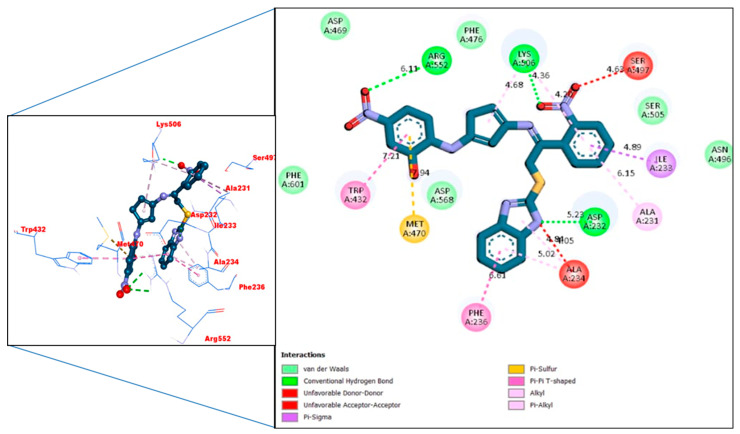
Protein–ligand interaction (PLI) analysis for the most potent compound **3** with the targeted BuChE enzyme, along with its 3D (**left**) and 2D (**right**) representations.

**Figure 8:**
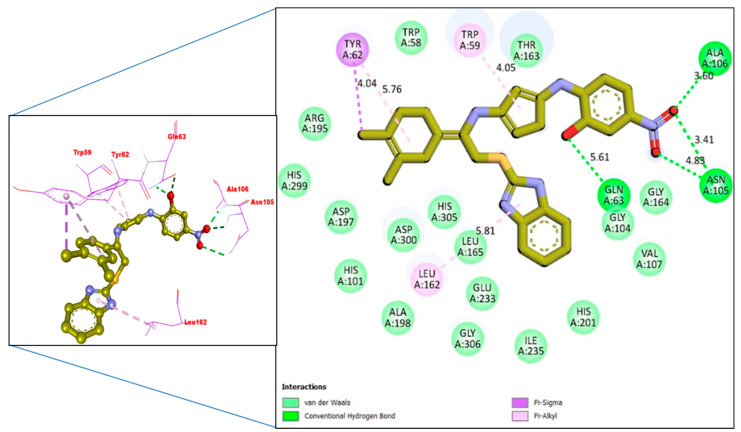
Protein–ligand interaction (PLI) analysis for the 2nd most potent compound **5** with the targeted BuChE enzyme, along with its 3D (**left**) and 2D (**right**) representations.

**Figure 9:**
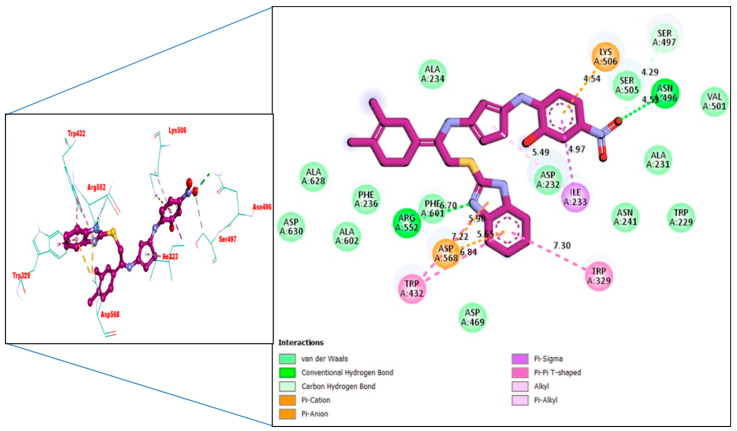
Protein–ligand interaction (PLI) analysis for the 2nd most potent compound **5** with the targeted BuChE enzyme, along with its 3D (**left**) and 2D (**right**) representations.

**Figure 10:**
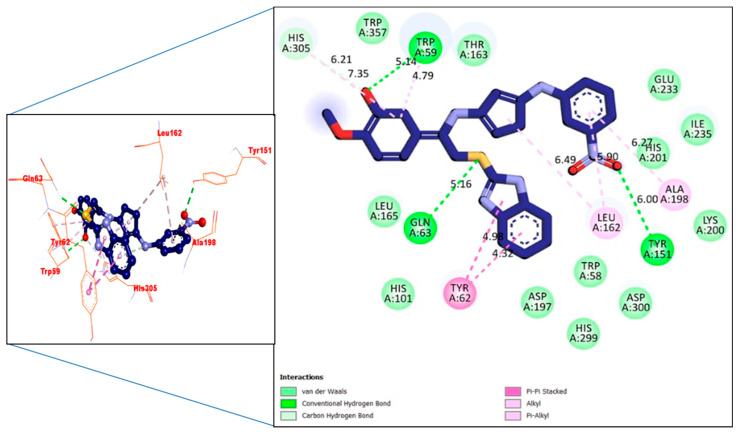
Protein–ligand interaction (PLI) analysis for the 3rd most potent compound **9** with the targeted AChE enzyme, along with its 3D (**left**) and 2D (**right**) representations.

**Figure 11:**
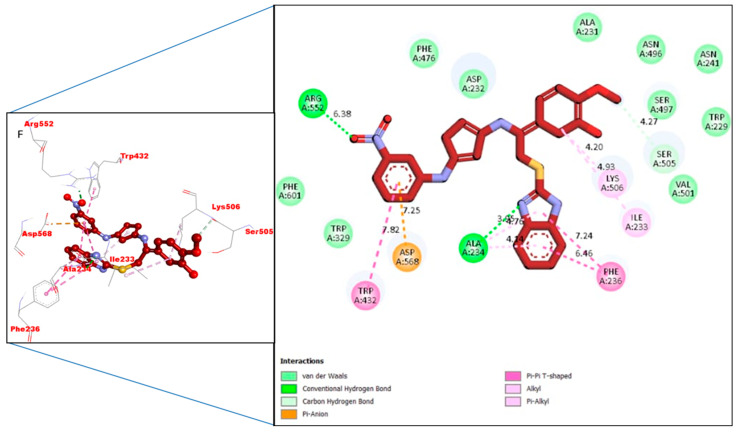
Protein–ligand interaction (PLI) analysis for the 3rd most potent compound **9** with the targeted BuChE enzyme, along with its 3D (**left**) and 2D (**right**) representations.

A docking analysis was conducted on the most potent analogs employing multiple software tools, including AutoDock (version 1.5.7), as well as Pymol and Discovery Studio Visualizer (DSV), for visualizing the 2D and 3D molecular structures within the protein complex. To establish a meaningful correlation among in vitro and in silico studies, we conducted docking analyses on the most potent scaffolds against AChE and BuChE. The purpose of this was to elucidate the interactions among the active scaffolds (scaffolds **3**, **5**, and **9**) and the active sites of the enzymes, which play a pivotal role in their functioning. These interactions were further substantiated by examining protein–ligand interactions (PLI), as outlined in detail in Table 2. Notably, the outcomes of these interactions, depicted in Figure 6, Figure 7, Figure 8, Figure 9, Figure 10 and Figure 11, underscore the significance of these scaffold molecules in augmenting the enzymatic activities of AChE and BuChE.

## 3. Materials and Methods

### 3.1. General Information 

All chemicals and reagents employed in the study were obtained from Merck or Sigma-Aldrich (St. Louis, MO, USA) (Darmstadt, Germany). Acetylcholinesterase (E.C.3.1.1.7) and butyrylcholinesterase (E.C. 3.1.1.8) were procured from Sigma–Aldrich (Steinheim, Germany). The ^1^H NMR and ^13^C NMR spectra were recorded using a Bruker FT-NMR spectrometer operating at the digital frequencies of 600 and 150 MHz (Bioscience, Bruker, Billerica, MA, USA). Mass spectra were acquired utilizing a Shimadzu LCMS-IT-TOF system (Kyoto, Japan) employing the electrospray ionization (ESI) technique. The purity of synthesized derivatives was checked through thin-layer chromatography using silica gel 60 F254 (Merck, KGaA, Darmstadt, Germany).

### 3.2. General Method for Synthesizing Benzimidazole-Based Schiff Base Derivatives (***1***–***18***)

#### 3.2.1. Procedure for 2-((1H-benzo[d]imidazol-2-yl)thio)-1-phenylethan-1-one (**III**)

The carbon disulphide (1 mmol) and benzene-1,2-diammine (**I**, 1 mmol) were taken in EtOH-H_2_O (10 mL). The mixture was agitated for a duration of 6 h, and the precipitates obtained were then subjected to a drying process and underwent recrystallization to yield an intermediate product (**II**). Then, an equimolar amount of substrate (**II**) and different substituted phenacyl bromide were refluxed and stirred in ethanol (10 mL) and triethylamine (catalyst) to afford S-substituted benzimidazole (**III**).

#### 3.2.2. Procedure for Targeted Benzimidazole-Based Schiff Base Analogues (**1**–**18**)

Lastly, in the synthesis process, S-substituted benzimidazole (**III**, 1 mmol) and various substituted N1-phenylcyclopenta-3,5-diene-1,3-diamine compounds (1 mmol) were mixed in 10 mL of glacial acetic acid and refluxed for a duration of 6 h and subsequently transferred into frigid water. The resultant solid was subjected to washing, drying, and subsequent recrystallization, ultimately resulting in the formation of the desired benzimidazole-based Schiff base analogs (**1**–**18**).

### 3.3. Structural Analysis

#### 3.3.1. (Z)-2-((4-((2-((1H-benzo[d]imidazol-2-yl)thio)-1-(4-nitrophenyl)ethylidene)amino)cyclopenta-1,3-dien-1-yl)amino)-5-nitrophenol (**1**)

Yield: 65%; yellow solid; m.p.: 192−193 °C; ^1^H-NMR (600 MHz, DMSO-d_6_): δ 13.14 (s, 1H, NH), 12.28 (s, 1H, NH), 9.68 (s, 1H, OH), 8.71 (d, 2H, J = 7.0 Hz, Ar-H (R), 8.64 (d, 2H, J = 7.6 Hz, Ar-H (R), 8.56 (d, J = 7.8 Hz, 1H, Ar-H (R_1_), 8.49 (d, J = 8.0 Hz, 2H, Benzimidazol-H), 8.44 (s, 1H, Ar-H (R_1_), 8.16 (t, J = 7.8 Hz, 2H, Benzimidazol-H), 8.09 (d, J = 7.7 Hz, 1H, Ar-H (R_1_), 6.21 (s, 1H, cyclopentene-H), 6.14 (s, 1H, cyclopentene-H), 3.85 (s, 2H, S-CH_2_), 3.01 (s, 2H, cyclopentene-H); ^13^C-NMR (150 MHz, DMSO-d_6_): δ 172.8, 161.6, 160.2 159.9, 158.2, 156.4, 153.1, 151.7, 151.3, 150.2, 149.9, 148.4, 146.2, 144.9, 143.2, 141.2, 137.7, 137.5, 133.4, 130.7, 128.1, 126.7, 118.0, 99.1, 56.3, 37.3; HREI-MS: *m*/*z* calcld for C_26_H_20_N_6_O_5_S, [M]^+^ 528.4873 Found 528.4824.

#### 3.3.2. (Z)-2-((4-((2-((1H-benzo[d]imidazol-2-yl)thio)-1-(2-methyl-4-nitrophenyl)ethylidene)amino)cyclopenta-1,3-dien-1-yl)amino)-5-nitrophenol (**2**)

Yield: 66%; white solid; m.p.: 196−197 °C; ^1^H-NMR (600 MHz, DMSO-d_6_): δ 13.19 (s, 1H, NH), 12.24 (s, 1H, NH), 9.69 (s, 1H, OH), 8.61 (s, 1H, Ar-H (R), 8.58 (d, 1H, J = 7.5 Hz, Ar-H (R), 8.52 (d, J = 7.3 Hz, 1H, Ar-H (R), 8.46 (d, J = 8.3 Hz, 2H, Benzimidazol-H), 8.42 (s, 1H, Ar-H (R_1_), 8.34 (d, J = 7.3 Hz, 1H, Ar-H (R_1_), 8.28 (d, J = 7.4 Hz, 1H, Ar-H (R_1_), 8.25 (t, J = 7.4 Hz, 2H, Benzimidazol-H), 6.14 (s, 1H, cyclopentene-H), 6.10 (s, 1H, cyclopentene-H), 3.55 (s, 2H, S-CH_2_), 3.11 (s, 2H, cyclopentene-H) 2.56 (s, 3H, -CH_3_); ^13^C-NMR (150 MHz, DMSO-d_6_): δ 172.1, 162.4, 162.1 151.1, 151.5, 149.9, 149.6, 136.1, 130.2, 128.7, 128.4, 128.3, 127.9, 127.9, 126.2, 120.9, 118.2, 118.0, 117.2, 116.8, 116.6, 115.7, 115.6, 115.0, 56.0, 40.0, 21.2;HREI-MS: *m*/*z* calcld for C_27_H_22_N_6_O_5_S, [M]^+^ 542.7091 Found 542.7068.

#### 3.3.3. (Z)-2-((4-((2-((1H-benzo[d]imidazol-2-yl)thio)-1-(2-nitrophenyl)ethylidene)amino)cyclopenta-1,3-dien-1-yl)amino)-5-nitrophenol (**3**)

Yield: 68%; yellow solid; m.p.: 198−199 °C; ^1^H-NMR (600 MHz, DMSO-d_6_): δ 13.10 (s, 1H, NH), 12.21 (s, 1H, NH), 9.65 (s, 1H, OH), 8.68 (dd, 1H, J = 7.5 Hz, Ar-H (R), 8.58 (dd, 1H, J = 7.7 Hz, Ar-H (R), 8.53 (t, J = 7.0 Hz, 1H, Ar-H (R), 8.47 (t, J = 7.2 Hz, 1H, Ar-H (R), 8.41 (d, J = 8.0 Hz, 2H, Benzimidazol-H), 8.43 (s, 1H, Ar-H (R_1_), 8.34 (d, J = 7.2 Hz, 1H, Ar-H (R_1_), 8.21 (d, J = 7.5 Hz, 1H, Ar-H (R_1_), 8.17 (t, J = 7.8 Hz, 2H, Benzimidazol-H), 6.15 (s, 1H, cyclopentene-H), 6.10 (s, 1H, cyclopentene-H), 3.51 (s, 2H, S-CH_2_), 3.19 (s, 2H, cyclopentene-H); ^13^C-NMR (150 MHz, DMSO-d_6_): δ 176.4, 167.2, 165.3 164.9, 163.2, 161.3, 159.4, 158.7, 155.8, 153.1, 152.7, 150.1, 149.3, 146.2, 139.1, 134.2, 131.6, 127.7, 125.2, 121.6, 120.5, 117.9, 115.4, 99.5, 58.4, 39.0; HREI-MS: *m*/*z* calcld for C_26_H_20_N_6_O_5_S, [M]^+^ 528.1034 Found 528.1018.

#### 3.3.4. (Z)-2-((4-((2-((1H-benzo[d]imidazol-2-yl)thio)-1-(p-tolyl)ethylidene)amino)cyclopenta-1,3-dien-1-yl)amino)-5-nitrophenol (**4**)

Yield: 58%; white solid; m.p.: 205−207 °C; ^1^H-NMR (600 MHz, DMSO-d_6_): δ 11.55 (s, 1H, NH), 11.15 (s, 1H, NH), 9.86 (s, 1H, OH), 8.30 (d, 2H, J = 7.4 Hz, Ar-H (R), 8.26 (d, 2H, J = 7.4 Hz, Ar-H (R), 8.15 (d, J = 7.8 Hz, 1H, Ar-H (R_1_), 7.99 (d, J = 8.2 Hz, 2H, Benzimidazol-H), 7.73 (s, 1H, Ar-H (R_1_), 7.64 (t, J = 7.2 Hz, 2H, Benzimidazol-H), 7.52 (d, J = 7.2 Hz, 1H, Ar-H (R_1_), 6.98 (s, 1H, cyclopentene-H), 6.74 (s, 1H, cyclopentene-H), 3.41 (s, 2H, S-CH_2_), 2.51 (s, 2H, cyclopentene-H), 1.90 (s, 3H, -CH_3_);^13^C-NMR (150 MHz, DMSO-d_6_): δ 165.9, 164.3, 162.7, 160.3, 159.4, 157.0, 135.9, 131.0, 130.6, 130.6, 130.2, 130.1, 129.4, 129.3, 128.6, 128.3, 128.2, 127.7, 120.6, 116.1, 115.9, 115.8, 115.8, 115.7, 64.8, 40.0, 21.0; HREI-MS: *m*/*z* calcld for C_27_H_23_N_5_O_3_S, [M]^+^ 497.5679 Found 497.5629.

#### 3.3.5. (Z)-2-((4-((2-((1H-benzo[d]imidazol-2-yl)thio)-1-(3,4-dichlorophenyl)ethylidene)amino)cyclopenta-1,3-dien-1-yl)amino)-5-nitrophenol (**5**)

Yield: 68%; brown solid; m.p.: 183−184 °C; ^1^H-NMR (600 MHz, DMSO-d_6_): δ 13.10 (s, 1H, NH), 12.27 (s, 1H, NH), 9.71 (s, 1H, OH), 8.62 (s, 1H, Ar-H (R), 8.59 (s, 1H, Ar-H (R_1_), 8.56 (d, 1H, J = 7.4 Hz, Ar-H (R), 8.53 (d, J = 7.2 Hz, 1H, Ar-H (R_1_), 8.50 (d, J = 7.4 Hz, 1H, Ar-H (R), 8.45 (d, J = 8.2 Hz, 2H, Benzimidazol-H), 8.30 (d, J = 7.0 Hz, 1H, Ar-H (R_1_), 8.23 (t, J = 7.2 Hz, 2H, Benzimidazol-H), 6.21 (s, 1H, cyclopentene-H), 6.17 (s, 1H, cyclopentene-H), 3.62 (s, 2H, S-CH_2_), 3.34 (s, 2H, cyclopentene-H); ^13^C-NMR (150 MHz, DMSO-d_6_): δ 174.1, 167.8, 164.2, 163.1, 162.9, 161.6, 159.2, 157.4, 156.6, 155.2, 154.7, 152.1, 147.2, 145.1, 141.6, 140.9, 138.7, 135.9, 133.4, 130.5, 126.5, 124.7, 121.5, 100.7, 56.3, 40.3; HREI-MS: *m*/*z* calcld for C_26_H_19_Cl_2_N_5_O_3_S, [M]^+^ 552.3931 Found 552.3908.

#### 3.3.6. (Z)-2-((4-((2-((1H-benzo[d]imidazol-2-yl)thio)-1-(2-bromo-4-nitrophenyl)ethylidene)amino)cyclopenta-1,3-dien-1-yl)amino)-5-nitrophenol (**6**)

Yield: 63%; light yellow solid; m.p.: 194−195 °C; ^1^H-NMR (600 MHz, DMSO-d_6_): δ 13.10 (s, 1H, NH), 12.08 (s, 1H, NH), 9.65 (s, 1H, OH), 8.62 (s, 1H, Ar-H (R), 8.59 (d, 1H, J = 7.4 Hz, Ar-H (R), 8.53 (d, J = 7.3 Hz, 1H, Ar-H (R), 8.47 (d, J = 8.2 Hz, 2H, Benzimidazol-H), 8.43 (d, J = 7.0 Hz, 1H, Ar-H (R_1_), 8.40 (s, 1H, Ar-H (R_1_), 8.29 (d, J = 7.5 Hz, 1H, Ar-H (R_1_), 8.23 (t, J = 7.5 Hz, 2H, Benzimidazol-H), 6.16 (s, 1H, cyclopentene-H), 6.11 (s, 1H, cyclopentene-H), 3.56 (s, 2H, S-CH_2_), 3.12 (s, 2H, cyclopentene-H); ^13^C-NMR (150 MHz, DMSO-d_6_): δ 171.4, 160.2, 159.3 157.2, 156.9, 155.2, 154.4, 152.0, 151.3, 151.0, 149.1, 147.1, 145.1, 143.9, 140.2, 138.2, 137.0, 134.9, 131.5, 128.4, 126.5, 125.7, 124.4, 101.4, 55.2; 38.7; HREI-MS: *m*/*z* calcld for C_26_H_19_BrN_6_O_5_S, [M]^+^ 607.3098 Found 607.3052.

#### 3.3.7. (Z)-2-((4-((2-((1H-benzo[d]imidazol-2-yl)thio)-1-(2-bromophenyl)ethylidene)amino)cyclopenta-1,3-dien-1-yl)amino)-5-nitrophenol (**7**)

Yield: 67%; black solid; m.p.: 190−191 °C; ^1^H-NMR (600 MHz, DMSO-d_6_): δ 13.11 (s, 1H, NH), 12.19 (s, 1H, NH), 9.61 (s, 1H, OH), 8.67 (dd, 1H, J = 7.0 Hz, Ar-H (R), 8.57 (dd, 1H, J = 7.9 Hz, Ar-H (R), 8.51 (t, J = 7.8 Hz, 1H, Ar-H (R), 8.45 (t, J = 7.1 Hz, 1H, Ar-H (R), 8.45 (d, J = 8.6 Hz, 2H, Benzimidazol-H), 8.41 (s, 1H, Ar-H (R_1_), 8.31 (d, J = 7.1 Hz, 1H, Ar-H (R_1_), 8.20 (d, J = 7.6 Hz, 1H, Ar-H (R_1_), 8.13 (t, J = 7.8 Hz, 2H, Benzimidazol-H), 6.10 (s, 1H, cyclopentene-H), 6.02 (s, 1H, cyclopentene-H), 3.55 (s, 2H, S-CH_2_), 3.27 (s, 2H, cyclopentene-H); ^13^C-NMR (150 MHz, DMSO-d_6_): δ 178.1, 170.6, 169.5 167.1, 164.2, 163.7, 161.4, 159.2, 157.8, 153.6, 152.2, 149.9, 149.0, 146.0, 139.7, 137.2, 136.6, 129.2, 126.2, 120.3, 120.0, 111.9, 109.2, 101.0, 59.1, 43.6; HREI-MS: *m*/*z* calcld for C_26_H_20_BrN_5_O_3_S, [M]^+^ 562.4204 Found 562.4188.

#### 3.3.8. (Z)-2-((4-((2-((1H-benzo[d]imidazol-2-yl)thio)-1-(o-tolyl)ethylidene)amino)cyclopenta-1,3-dien-1-yl)amino)-5-nitrophenol (**8**)

Yield: 54%; white solid; m.p.: 188−189 °C; ^1^H-NMR (600 MHz, DMSO-d_6_): δ 12.98 (s, 1H, NH), 12.14 (s, 1H, NH), 9.69 (s, 1H, OH), 8.72 (dd, 1H, J = 7.1 Hz, Ar-H (R), 8.66 (s, 1H, Ar-H (R_1_), 8.64 (d, J = 7.8 Hz, 1H, Ar-H (R_1_), 8.61 (d, J = 8.0 Hz, 2H, Benzimidazol-H), 8.57 (dd, 1H, J = 7.0 Hz, Ar-H (R), 8.45 (t, J = 7.2 Hz, 1H, Ar-H (R), 8.40 (t, J = 7.8 Hz, 1H, Ar-H (R), 8.21 (d, J = 7.9 Hz, 1H, Ar-H (R_1_), 7.98 (t, J = 7.8 Hz, 2H, Benzimidazol-H), 6.17 (s, 1H, cyclopentene-H), 6.13 (s, 1H, cyclopentene-H), 3.50 (s, 2H, S-CH_2_), 3.26 (s, 2H, cyclopentene-H), 2.41 (s, 3H, -CH_3_); ^13^C-NMR (150 MHz, DMSO-d_6_): δ159.4, 159.3, 158.2 153.9, 138.1, 135.7, 134.6, 133.9, 133.1, 132.2, 131.4, 130.7, 130.4, 130.3, 129.9, 129.4, 128.9, 128.7, 128.3, 127.9, 127.5, 126.2, 120.7, 115.7, 64.8, 40.0, 21.0; HREI-MS: *m*/*z* calcld for C_27_H_23_N_5_O_3_S, [M]^+^ 497.1185 Found 497.1148.

#### 3.3.9. (Z)-5-(2-((1H-benzo[d]imidazol-2-yl)thio)-1-((4-((3-nitrophenyl)amino)cyclopenta-1,3-dien-1-yl)imino)ethyl)-2-methoxyphenol (**9**)

Yield: 59%; white solid; m.p.: 186−187 °C; ^1^H-NMR (600 MHz, DMSO-d_6_): δ 13.11 (s, 1H, NH), 12.13 (s, 1H, NH), 9.87 (s, 1H, -OH), 8.63 (s, 1H, Ar-H (R_1_), 8.61 (dd, 1H, J = 7.2, 2.1 Hz, Ar-H (R_1_), 8.54 (dd, J = 8.0, 2.6 Hz, 2H, Benzimidazol-H), 8.49 (t, J = 7.6 Hz, 1H, Ar-H (R_1_), 8.44 (s, 1H, Ar-H (R), 8.43 (d, J = 7.2 Hz, 1H, Ar-H (R), 8.26 (t, J = 7.8 Hz, 2H, Benzimidazol-H), 8.03 (dd, J = 7.8, 2.0 Hz, 1H, Ar-H (R_1_), 7.97 (d, J = 7.2 Hz, 1H, Ar-H (R), 6.09 (s, 1H, cyclopentene-H), 6.05 (s, 1H, cyclopentene-H), 3.85 (s, 3H, -OCH_3_), 3.57 (s, 2H, S-CH_2_), 3.13 (s, 2H, cyclopentene-H); ^13^C-NMR (150 MHz, DMSO-d_6_): δ 176.4, 164.2, 162.5 160.2, 159.9, 158.2, 157.4, 155.0, 154.3, 151.4, 148.2, 146.2, 145.4, 142.9, 139.2, 136.2, 134.0, 131.9, 130.5, 129.4, 127.5, 125.5, 121.7, 100.3, 56.2; 39.7, 31.4; HREI-MS: *m*/*z* calcld for C_27_H_23_N_5_O_4_S, [M]^+^ 513.2165 Found 513.2142.

#### 3.3.10. (Z)-2-((4-((2-((1H-benzo[d]imidazol-2-yl)thio)-1-phenylethylidene)amino)cyclopenta-1,3-dien-1-yl)amino)-5-nitrophenol (**10**)

Yield: 56%; black solid; m.p.: 201−202 °C; ^1^H-NMR (600 MHz, DMSO-d_6_): δ 13.08 (s, 1H, NH), 12.09 (s, 1H, NH), 9.76 (s, 1H, OH), 8.70 (dd, 2H, J = 7.1 Hz, Ar-H (R), 8.61 (t, 2H, J = 7.3 Hz, Ar-H (R), 8.54 (m, J = 7.0 Hz, 1H, Ar-H (R), 8.41 (d, J = 8.7 Hz, 2H, Benzimidazol-H), 8.36 (s, 1H, Ar-H (R_1_), 8.34 (d, J = 7.0 Hz, 1H, Ar-H (R_1_), 8.19 (d, J = 7.3 Hz, 1H, Ar-H (R_1_), 7.91 (t, J = 7.9 Hz, 2H, Benzimidazol-H), 6.16 (s, 1H, cyclopentene-H), 6.12 (s, 1H, cyclopentene-H), 3.58 (s, 2H, S-CH_2_), 3.33 (s, 2H, cyclopentene-H); ^13^C-NMR (150 MHz, DMSO-d_6_): δ 170.8, 161.5, 160.3 160.0, 155.2, 154.7, 153.2, 152.8, 150.8, 149.1, 148.4, 143.0, 140.3, 138.0, 137.7, 136.2, 132.3, 129.4, 126.8, 125.8, 122.4, 119.1, 113.5, 100.0, 61.5, 44.8; HREI-MS: *m*/*z* calcld for C_26_H_21_N_5_O_3_S, [M]^+^ 483.0183 Found 483.0154.

#### 3.3.11. (Z)-N-(4-((2-((1H-benzo[d]imidazol-2-yl)thio)-1-(4-nitrophenyl)ethylidene)amino)cyclopenta-1,3-dien-1-yl)-3-nitroaniline (**11**)

Yield: 68%; green solid; m.p.: 182−184 °C; ^1^H-NMR (600 MHz, DMSO-d_6_): δ 13.19 (s, 1H, NH), 12.21 (s, 1H, NH), 8.77 (d, J = 7.9 Hz, 2H, Ar-H (R), 8.63 (d, J = 7.0 Hz, 2H, Ar-H (R), 8.60 (s, 1H, Ar-H (R_1_), 8.57 (dd, 1H, J = 7.3, 2.5 Hz, Ar-H (R_1_), 8.50 (dd, J = 7.9, 1.8 Hz, 2H, Benzimidazol-H), 8.38 (t, J = 7.8 Hz, 1H, Ar-H (R_1_), 8.23 (t, J = 7.9 Hz, 2H, Benzimidazol-H), 8.17 (dd, J = 7.0, 2.1 Hz, 1H, Ar-H (R_1_), 6.18 (s, 1H, cyclopentene-H), 6.13 (s, 1H, cyclopentene-H), 3.62 (s, 2H, S-CH_2_), 3.23 (s, 2H, cyclopentene-H); ^13^C-NMR (150 MHz, DMSO-d_6_): δ 178.1, 171.0, 170.7, 157.2, 156.4, 154.7, 151.4, 150.0, 149.3, 141.4, 140.2, 136.2, 135.7, 132.1, 129.1, 126.6, 117.0, 116.9, 113.4, 112.3, 111.5, 110.5, 110.2, 100.3, 61.2; 43.5; HREI-MS: *m*/*z* calcld for C_26_H_20_N_6_O_4_S, [M]^+^ 512.1176 Found 512.1147.

#### 3.3.12. (Z)-N-(4-((2-((1H-benzo[d]imidazol-2-yl)thio)-1-(p-tolyl)ethylidene)amino)cyclopenta-1,3-dien-1-yl)-3-nitroaniline (**12**)

Yield: 57%; green solid; m.p.: 186−188 °C; ^1^H-NMR (600 MHz, DMSO-d_6_): δ 13.10 (s, 1H, NH), 12.14 (s, 1H, NH), 8.65 (s, 1H, Ar-H (R_1_), 8.62 (d, J = 7.3 Hz, 2H, Ar-H (R), 8.57 (d, J = 7.2 Hz, 2H, Ar-H (R), 8.48 (dd, 1H, J = 7.4, 2.1 Hz, Ar-H (R_1_), 8.37 (dd, J = 8.0, 1.4 Hz, 2H, Benzimidazol-H), 8.24 (t, J = 7.0 Hz, 1H, Ar-H (R_1_), 8.11(t, J = 7.4 Hz, 2H, Benzimidazol-H), 7.97 (dd, J = 7.4, 2.5 Hz, 1H, Ar-H (R_1_), 6.14 (s, 1H, cyclopentene-H), 6.09 (s, 1H, cyclopentene-H), 3.61 (s, 2H, S-CH_2_), 3.29 (s, 2H, cyclopentene-H), 2.65 (s, 3H, -CH_3_); ^13^C-NMR (150 MHz, DMSO-d_6_): δ 177.8, 170.2, 169.4, 156.6, 156.3, 155.7, 154.4, 153.0, 151.1, 149.8, 147.2, 146.8, 145.7, 1441, 139.1, 136.2, 127.0, 126.2, 123.5, 122.4, 119.1, 117.3, 111.2, 99.3, 63.2; 45.6, 32.5; HREI-MS: *m*/*z* calcld for C_27_H_23_N_5_O_2_S, [M]^+^ 481.1079 Found 481.1046.

#### 3.3.13. (Z)-N-(4-((2-((1H-benzo[d]imidazol-2-yl)thio)-1-(m-tolyl)ethylidene)amino)cyclopenta-1,3-dien-1-yl)-3-nitroaniline (**13**)

Yield: 65%; orange solid; m.p.: 202−204 °C; ^1^H-NMR (600 MHz, DMSO-d_6_): δ13.38 (s, 1H, NH), 11.27 (s, 1H, NH), 8.74 (s, 1H, Ar-H (R_1_), 8.69 (s, 1H, Ar-H (R), 8.37 (dd, J = 8.1, 2.2 Hz, 2H, Benzimidazol-H), 8.19 (t, J = 7.4 Hz, 2H, Benzimidazol-H), 7.96 (dd, J = 7.4, 1.9 Hz, 1H, Ar-H (R), 7.74 (dd, J = 7.2, 1.7 Hz, 1H, Ar-H (R), 7.56 (dd, 1H, J = 7.9, 2.6 Hz, Ar-H (R_1_), 7.34 (t, J = 6.8 Hz, 1H, Ar-H (R_1_), 7.12 (t, J = 7.1 Hz, 1H, Ar-H (R), 7.09 (dd, J = 7.8, 1.9 Hz, 1H, Ar-H (R_1_), 6.98 (s, 1H, cyclopentene-H), 6.97 (s, 1H, cyclopentene-H), 3.93 (s, 2H, S-CH_2_), 2.08 (s, 2H, cyclopentene-H), 1.23(s, 3H, -CH_3_); ^13^C-NMR (150 MHz, DMSO-d_6_): δ 172.8, 167.2, 166.4, 152.1, 151.3, 150.6, 150.3, 149.2, 147.3, 146.8, 146.1, 144.8, 144.4, 140.1, 136.5, 135.2, 133.4, 131.9, 128.4, 126.4, 116.3, 112.4, 111.9, 99.7, 65.2; 46.2, 33.9; HREI-MS: *m*/*z* calcld for C_27_H_23_N_5_O_2_S, [M]^+^ 481.1798 Found 481.1762.

#### 3.3.14. (Z)-N-(4-((2-((1H-benzo[d]imidazol-2-yl)thio)-1-(o-tolyl)ethylidene)amino)cyclopenta-1,3-dien-1-yl)-3-nitroaniline (**14**)

Yield: 62%; orange solid; m.p.: 199−201 °C; ^1^H-NMR (600 MHz, DMSO-d_6_): δ 13.09 (s, 1H, NH), 12.16 (s, 1H, NH), 8.71 (dd, 1H, J = 7.9, 2.3 Hz, Ar-H (R), 8.69 (t, 1H, J = 7.4 Hz, Ar-H (R), 8.62 (t, J = 7.0 Hz, 1H, Ar-H (R), 8.51 (d, J = 8.4 Hz, 2H, Benzimidazol-H), 8.37 (s, 1H, Ar-H (R_1_), 8.28 (d, J = 7.5 Hz, 1H, Ar-H (R_1_), 8.15 (dd, J = 7.4, 2.4 Hz, 1H, Ar-H (R), 7.94 (t, J = 7.7 Hz, 2H, Benzimidazol-H), 7.84 (dd, 1H, J = 7.0, 1.8 Hz, Ar-H (R_1_), 6.15 (s, 1H, cyclopentene-H), 6.11 (s, 1H, cyclopentene-H), 3.56 (s, 2H, S-CH_2_), 3.32 (s, 2H, cyclopentene-H), 2.63 (s, 3H, -CH_3_); ^13^C-NMR (150 MHz, DMSO-d_6_): δ 171.2, 162.4, 161.2 160.0, 159.4, 155.2, 154.9, 153.9, 149.8, 149.0, 144.4, 140.0, 139.7, 135.8, 133.2, 132.6, 129.3, 129.0, 123.7, 121.1, 118.5, 114.1, 112.4, 100.2, 60.4, 45.8, 30.4; HREI-MS: *m*/*z* calcld for C_27_H_23_N_5_O_2_S, [M]^+^ 481.6103 Found 481.6144.

#### 3.3.15. (Z)-N-(4-((2-((1H-benzo[d]imidazol-2-yl)thio)-1-(2-methyl-4-nitrophenyl)ethylidene)amino)cyclopenta-1,3-dien-1-yl)-3-nitroaniline (**15**)

Yield: 63%; red solid; m.p.: 184−186 °C; ^1^H-NMR (600 MHz, DMSO-d_6_): δ 13.13 (s, 1H, NH), 12.10 (s, 1H, NH), 8.65 (d, J = 7.9 Hz, 1H, Ar-H (R), 8.61 (s, 1H, Ar-H (R), 8.57 (d, J = 7.9 Hz, 1H, Ar-H (R), 8.48 (s, 1H, Ar-H (R_1_), 8.46 (dd, 1H, J = 7.3, 1.8 Hz, Ar-H (R_1_), 8.39 (dd, J = 7.4, 2.2 Hz, 2H, Benzimidazol-H), 8.32 (t, J = 7.8 Hz, 1H, Ar-H (R_1_), 8.24 (t, J = 7.0 Hz, 2H, Benzimidazol-H), 8.03 (dd, J = 7.8, 2.0 Hz, 1H, Ar-H (R_1_), 6.13 (s, 1H, cyclopentene-H), 6.09 (s, 1H, cyclopentene-H), 3.62 (s, 2H, S-CH_2_), 3.09 (s, 2H, cyclopentene-H), 2.45 (s, 3H, -CH_3_); ^13^C-NMR (150 MHz, DMSO-d_6_): δ 172.8, 161.8, 160.5 159.3, 159.0, 158.3, 154.2, 153.0, 150.3, 147.8, 143.5, 140.2, 136.4, 130.9, 128.2, 126.8, 124.0, 121.0, 120.5, 119.6, 117.1, 115.1, 114.2, 104.0, 40.8; 36.3, 28.0; HREI-MS: *m*/*z* calcld for C_27_H_22_N_6_O_4_S, [M]^+^ 526.0178 Found 526.0152.

#### 3.3.16. (Z)-N-(4-((2-((1H-benzo[d]imidazol-2-yl)thio)-1-(2,4-dichlorophenyl)ethylidene)amino)cyclopenta-1,3-dien-1-yl)-3-nitroaniline (**16**)

Yield: 68%; white solid; m.p.: 191−195 °C; ^1^H-NMR (600 MHz, DMSO-d_6_): δ 13.00 (s, 1H, NH), 12.13 (s, 1H, NH), 8.61 (s, 1H, Ar-H (R), 8.58 (s, 1H, Ar-H (R_1_), 8.43 (d, J = 7.6, 1H, Ar-H (R), 8.37 (dd, 1H, J = 7.0, 2.2 Hz, Ar-H (R_1_), 8.27 (d, J = 7.4 Hz, 1H, Ar-H (R), 8.16 (dd, J = 7.9, 2.0 Hz, 2H, Benzimidazol-H), 8.12 (t, J = 7.0 Hz, 1H, Ar-H (R_1_), 7.94 (t, J = 7.8 Hz, 2H, Benzimidazol-H), 7.81 (dd, J = 7.3, 1.8 Hz, 1H, Ar-H (R_1_), 6.11 (s, 1H, cyclopentene-H), 6.07 (s, 1H, cyclopentene-H), 3.68 (s, 2H, S-CH_2_), 2.97 (s, 2H, cyclopentene-H); ^13^C-NMR (150 MHz, DMSO-d_6_): δ 170.4, 160.9, 160.1 159.4, 159.2, 157.3, 155.2, 155.0, 152.3, 151.8, 151.5, 150.2, 144.6, 140.5, 138.4, 134.8, 132.0, 131.0, 130.5, 129.4, 127.6, 119.1, 117.8, 105.2, 40.8, 28.5; HREI-MS: *m*/*z* calcld for C_26_H_19_Cl_2_N_5_O_2_S, [M]^+^ 536.7129 Found 536.7101.

#### 3.3.17. (Z)-N-(4-((2-((1H-benzo[d]imidazol-2-yl)thio)-1-(2-bromophenyl)ethylidene)amino)cyclopenta-1,3-dien-1-yl)-3-nitroaniline (**17**)

Yield: 60%; brown solid; m.p.: 205−207 °C; ^1^H-NMR (600 MHz, DMSO-d_6_): δ 13.07 (s, 1H, NH), 12.09 (s, 1H, NH), 8.71 (s, 1H, Ar-H (R_1_), 8.62 (dd, 1H, J = 7.9, 2.3 Hz, Ar-H (R), 8.56 (t, 1H, J = 7.4 Hz, Ar-H (R), 8.44 (d, J = 8.0 Hz, 2H, Benzimidazol-H), 8.38 (t, J = 7.3 Hz, 1H, Ar-H (R), 8.30 (dd, J = 7.5, 1.3 Hz, 1H, Ar-H (R_1_), 8.05 (dd, J = 7.0, 2.1 Hz, 1H, Ar-H (R), 7.99 (t, J = 7.4 Hz, 1H, Ar-H (R_1_), 7.86 (t, J = 7.5Hz, 2H, Benzimidazol-H), 7.66 (dd, 1H, J = 7.0, 1.8 Hz, Ar-H (R_1_), 6.14 (s, 1H, cyclopentene-H), 6.12 (s, 1H, cyclopentene-H), 3.55 (s, 2H, S-CH_2_), 3.36 (s, 2H, cyclopentene-H); ^13^C-NMR (150 MHz, DMSO-d_6_): δ 172.2, 164.6, 162.6 161.4 157.8, 150.2, 149.9, 148.9, 147.3, 146.5, 145.4, 143.0, 139.8, 138.8, 136.3, 131.7, 126.4, 123.6, 121.7, 121.4, 117.5, 116.3, 114.4, 100.9, 53.8, 42.9; HREI-MS: *m*/*z* calcld for C_26_H_20_BrN_5_O_2_S, [M]^+^ 546.4105 Found 546.4384.

#### 3.3.18. (Z)-N-(4-((2-((1H-benzo[d]imidazol-2-yl)thio)-1-(2-bromo-4-nitrophenyl)ethylidene)amino)cyclopenta-1,3-dien-1-yl)-3-nitroaniline (**18**)

Yield: 66%; dark brown solid; m.p.: 190−192 °C; ^1^H-NMR (600 MHz, DMSO-d_6_): δ13.31 (s, 1H, NH), 11.26 (s, 1H, NH), 7.69 (s, 1H, Ar-H (R), 7.63 (d, J = 7.4, 1H, Ar-H (R), 7.55 (d, J = 7.8 Hz, 1H, Ar-H (R), 7.34 (s, 1H, Ar-H (R_1_), 7.11(t, J = 7.8 Hz, 4H, Benzimidazol-H), 7.08 (dd, 1H, J = 7.8, 2.1 Hz, Ar-H (R_1_), 6.97 (t, J = 7.7 Hz, 1H, Ar-H (R_1_), 6.90 (dd, J = 7.0, 1.8 Hz, 1H, Ar-H (R_1_), 6.89 (s, 1H, cyclopentene-H), 6.88 (s, 1H, cyclopentene-H), 3.88 (s, 2H, S-CH_2_), 2.49 (s, 2H, cyclopentene-H);^13^C-NMR (150 MHz, DMSO-d_6_): δ 169.1, 157.6, 150.7, 148.1, 147.8, 145.6, 143.0, 142.5, 140.4, 139.5, 139.3, 138.7, 138.5, 137.2, 134.0, 132.2, 131.4, 131.1, 128.5, 123.7, 120.1, 117.2, 113.3, 102.4, 40.61, 30.1; HREI-MS: *m*/*z* calcld for C_26_H_19_BrN_6_O_4_S, [M]^+^ 591.1357 Found 591.1341.

### 3.4. Acetylcholinesterase and Butyrylcholinesterase Inhibition Assay

The study was carried out following the guidelines outlined in our previously published study [29].

### 3.5. Molecular Docking Protocol

The study was carried out following the guidelines outlined in our previously published study [30,31,32].

## 4. Conclusions

In conclusion, we have focused our efforts on reporting benzimidazole-based Schiff base derivatives (**1**–**18**) for their anti-Alzheimer characteristics. To evaluate their capacity as anti-Alzheimer agents, all these derivatives (**1**–**18**) were examined for in vitro assays targeting acetylcholinesterase (AChE) and butyrylcholinesterase (BuChE). An analysis of the structure–activity relationships indicated that the variations in activities against both enzymes were attributed to specific substitution patterns of substituents at various positions on the aryl rings. The anti-Alzheimer assay results showcased notable inhibition, with IC_50_ values spanning from 123.9 ± 10.20 to 342.60 ± 10.60 µM (against AChE) and 131.30 ± 9.70 to 375.80 ± 12.80 µM (against BuChE). Notably, compounds **3**, **5**, and **9** displayed exceptional activity, outperforming the standard inhibitor Donepezil, with IC_50_ values of 123.90 ± 10.20, 168.60 ± 7.70, and 174.22 ± 6.76 (against AChE) and 131.30 ± 9.70, 179.30 ± 6.70, and 181.20 ± 8.50 (against BuChE), respectively.

These findings underscore the potential of benzimidazole–Schiff-base scaffolds as AChE and BuChE inhibitors, holding potential for advancing innovative treatments for Alzheimer’s disease. Noteworthy analogues, particularly **3**, **5**, and **9**, exhibit significant potency and hold potential as lead compounds in the quest for pioneering anti-Alzheimer agents.

## Figures and Tables

**Table 1:** Structures and in vitro inhibitory effects of benzimidazole-based Schiff base derivatives against acetylcholinesterase (AChE) and butyrylcholinesterase (BuChE) (**1**–**18**).

Compounds	R	R_1_	AChE Inhibition IC_50_ ± SEM ^a^ [µM]	BuChE Inhibition IC_50_ ± SEM ^a^ [µM]
**1**	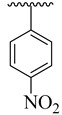	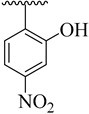	213.78 ± 11.60	248.70 ± 10.90
**2**	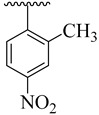	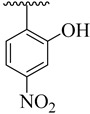	198.82 ± 10.20	203.70 ± 8.20
**3**	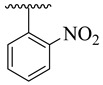	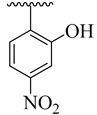	123.90 ± 10.20	131.30 ± 9.70
**4**	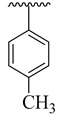	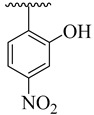	273.50 ± 7.60	285.32 ± 8.20
**5**	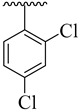	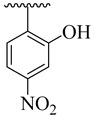	168.60 ± 7.70	179.30 ± 6.70
**6**	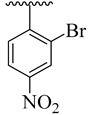	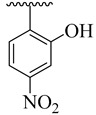	226.30 ± 10.10	273.70 ± 13.80
**7**	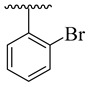	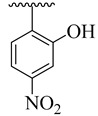	313.60 ± 12.80	325.80 ± 13.10
**8**	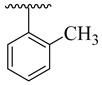	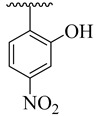	290.70 ± 12.60	303.60 ±13.80
**9**	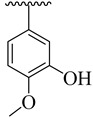	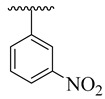	174.22 ± 6.76	181.20 ± 8.50
**10**	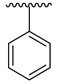	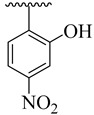	342.60 ± 10.60	375.80 ± 12.80
**11**	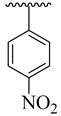	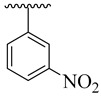	267.50 ± 11.70	273.60 ± 9.10
**12**	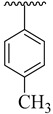	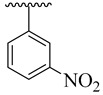	308.40 ± 8.80	317.68 ± 9.80
**13**	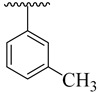	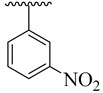	320.67 ± 13.50	354.10 ± 12.70
**14**	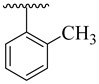	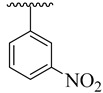	288.90 ± 11.50	310.70 ± 12.30
**15**	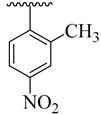	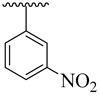	216.70 ± 8.28	221.34 ± 8.70
**16**	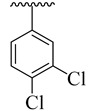	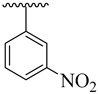	209.45 ± 8.30	218.50 ± 8.50
**17**	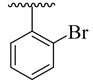	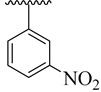	233.82 ± 10.10	269.40 ± 9.60
**18**	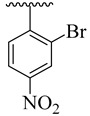	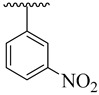	213.50 ± 8.20	246.60 ± 9.10
Standard Donepezil drug			243.76 ± 5.70	276.60 ± 6.50

^a^ Standard error mean (SEM).

**Table 2:** The interaction details between ligands and active residues of targeted enzymes, along with docking scores.

Active Analogues	Distance (A°)	Types of Interactions	Receptor	Docking Score
**Compound-3** **AChE** complex	5.2	H-B	HIS-A-101	−10.20
	4.96	Pi-Pi stacked	TYR-A-62	
	5.14	Pi-Sigma	LEU-A-162	
	5.15	Pi-R	LEU-A-162	
	6.14	Pi-R	ALA-A-198	
	6.75	Pi-cation	HIS-A-201	
	6.13	H-B	GLN-A-63	
	3.48	H-B	ASN-A-105	
	3.58	H-B	ALA-A-106	
	4.19	Pi-R	TRP-A-59	
**Compound-3** in **BuChE** complex	4.94	Pi-S	MET-A-470	−11.70
	7.21	Pi-Pi T-shaped	TRP-A-432	
	6.11	H-B	ASN-A-552	
	4.68	Pi-R	LYS-A-506	
	4.36	H-B	LYS-A-506	
	4.26	Pi-R	LYS-A-506	
	4.63	Unfavorable D-D	SER-A-497	
	4.89	Pi-sigma	ILE-A-233	
	6.15	Pi-R	ALA-A-231	
	5.23	H-B	ASP-A-232	
	4.05	Unfavorable D-D	ALA-A-234	
	4.94	Pi-R	ALA-A-234	
	5.02	Pi-R	ALA-A-234	
	6.01	Pi-Pi T-shaped	PHE-A-236	
**Compound-5** In **AChE** complex	5.81	Pi-R	LEU-A-162	−9.12
	4.04	Pi-sigma	TYR-A-62	
	5.76	Pi-R	TYR-A-62	
	4.05	Pi-R	TRP-A-59	
	3.6	H-B	ALA-A-106	
	3.41	H-B	ASN-A-105	
	4.83	H-B	ASN-A-105	
	5.61	H-B	GLN-A-63	
**Compound-5** in **BuChE** complex	6.84	Pi-Pi T-shaped	TRP-A-432	−11.40
	5.45	Pi-Pi T-shaped	TRP-A-432	
	7.22	Pi-cation	ASP-A-568	
	5.98	Pi-cation	ASP-A-568	
	6.7	H-B	PHE-A-601	
	4.54	Pi-cation	LYS-A-506	
	4.29	Pi-R	SER-A-497	
	4.54	H-B	ASN-A-496	
	4.97	Pi-sigma	ILE-A-233	
	5.49	Pi-sigma	ASP-A-232	
	7.3	Pi-Pi T-shaped	TRP-A-329	
**Compound-9** in **AChE** complex	4.32	Pi-Pi Stacked	TYR-A-62	−11.28
	4.94	Pi-Pi Stacked	TYR-A-62	
	5.16	H-B	GLN-A-63	
	6.21	Pi-R	HIS-A-305	
	7.35	Pi-R	TRP-A-59	
	5.14	H-B	TRP-A-357	
	4.79	Pi-R	TRP-A-59	
	6.27	Pi-R	ALA-A-198	
	6	H-B	TYR-A-151	
	5.9	Pi-R	LEU-A-162	
	6.49	Pi-R	LEU-A-162	
**Compound-9** in **BuChE** complex	7.25	Pi-anion	ASP-A-568	−10.50
	7.82	Pi-Pi Stacked	TRP-A-357	
	6.38	H-B	ARG-A-552	
	4.27	C-H	SER-A-505	
	4.2	Pi-R	LYS-A-506	
	4.93	Pi-R	ILE-A-233	
	7.24	Pi-Pi Stacked	PHE-A-236	
	6.46	Pi-Pi Stacked	PHE-A-236	
	4.74	Pi-R	ALA-A-234	
	4.76	Pi-R	ALA-A-234	
	3.95	H-B	ALA-A-234	

## Data Availability

Data is contained within the article and supplementary material.

## Supplementary Materials

The following supporting information can be downloaded at: https://www.mdpi.com/article/10.3390/ph16091278/s1, Flow Chart representation of the Scheme 1. Figure S1. ^13^CNMR for the compound **2** *(Z)-2-((4-((2-((1H-benzo[d]imidazol-2-yl)thio)-1-(2-methyl-4-nitrophenyl)ethylidene)amino)cyclopenta-1,3-dien-1-yl)amino)-5-nitrophenol* (**2**). Figure S2. ^1^HNMR for the compound **4** *(Z)-2-((4-((2-((1H-benzo[d]imidazol-2-yl)thio)-1-(p-tolyl)ethylidene)amino)cyclopenta-1,3-dien-1-yl)amino)-5-nitrophenol* (**4**). Figure S3. ^13^CNMR for the compound **4** *(Z)-2-((4-((2-((1H-benzo[d]imidazol-2-yl)thio)-1-(p-tolyl)ethylidene)amino)cyclopenta-1,3-dien-1-yl)amino)-5-nitrophenol* (**4**). Figure S4. ^13^CNMR for the compound **8** *(Z)-2-((4-((2-((1H-benzo[d]imidazol-2-yl)thio)-1-(o-tolyl)ethylidene)amino)cyclopenta-1,3-dien-1-yl)amino)-5-nitrophenol* (**8**). Figure S5. ^1^HNMR for the compound **13** *(Z)-N-(4-((2-((1H-benzo[d]imidazol-2-yl)thio)-1-(m-tolyl)ethylidene)amino)cyclopenta-1,3-dien-1-yl)-3-nitroaniline* (**13**). Figure S6. ^1^HNMR for the compound **18** *(Z)-N-(4-((2-((1H-benzo[d]imidazol-2-yl)thio)-1-(2-bromo-4-nitrophenyl)ethylidene)amino)cyclopenta-1,3-dien-1-yl)-3-nitroaniline* (**18**).
