# Supplementary material for: Benzimidazole-Based Schiff Base Hybrid Scaffolds: A Promising Approach to Develop Multi-Target Drugs for Alzheimer’s Disease
Source: Pharmaceuticals (Basel). 2023 Sep 11;16(9):1278. doi: 10.3390/ph16091278 (PMC10535318; doi:10.3390/ph16091278)
Supplement: Supplementary file 1 [file pharmaceuticals-16-01278-s001.zip › pharmaceuticals-2534263-supplementary.pdf]

Benzimidazole-Based Schiff Base Hybrids Scaffolds: A Promising Approach to Develop Multi-Target Drugs for Alzheimer's Disease

Rafaqat Hussain [1], Shoaib Khan [2,*], Hayat Ullah [3,*], Farhan Ali [2], Yousaf Khan [4], Asma Sardar [1], Rashid Iqbal [5,6], Farid S. Ataya [7], Nasser M. El-Sabbagh [8] and Gaber El-Saber Batiha [9]

[1] Department of Chemistry, Hazara University, Mansehra 21120, Pakistan; rafaqathussain0347@gmail.com (R.H.); asmasardar@gmail.com (A.S.)
[2] Department of Chemistry, Abbottabad University of Science and Technology (AUST), Abbottabad 22020, Pakistan
[3] Department of Chemistry, University of Okara, Okara 56130, Pakistan
[4] Department of Chemistry, COMSATS University, Islamabad 45550, Pakistan
[5] Department of Agroecology-Climate and Water, Aarhus University, Blichers Allé 20, 8830 Tjele, Denmark; rashid.iqbal@iub.edu.pk
[6] Department of Agronomy, Faculty of Agriculture and Environment, The Islamia University of Bahawalpur, Bahawalpur 63100, Pakistan
[7] Department of Biochemistry, College of Science, King Saud University, P.O. Box 2455, Riyadh 11451, Saudi Arabia; fataya@ksu.edu.sa
[8] Department of Veterinary Pharmacology, Faculty of Veterinary Medicine, Alexandria University, Alexandria 21526, Egypt; nasserelsabbagh@yahoo.com
[9] Department of Pharmacology and Therapeutics, Faculty of Veterinary Medicine, Damanhour University, Damanhour 22511, AlBeheira, Egypt; dr_gaber_batiha@vetmed.dmu.edu.eg
* Correspondence: shoaibkhanswati@gmail.com (S.K.); ayaanwazir366@gmail.com (H.U.)

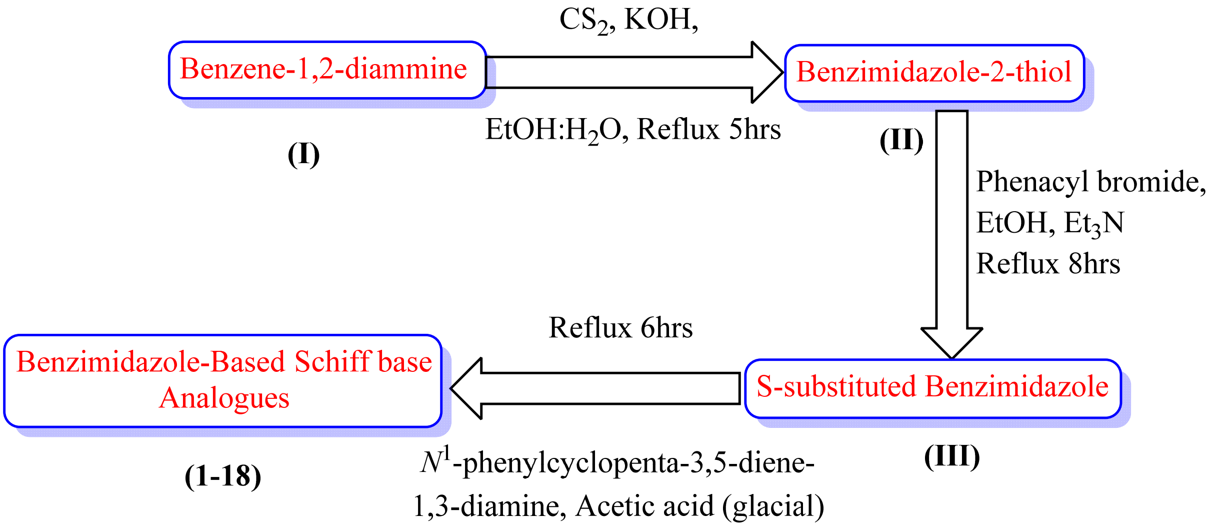

Flow Chart representation of the Scheme 1

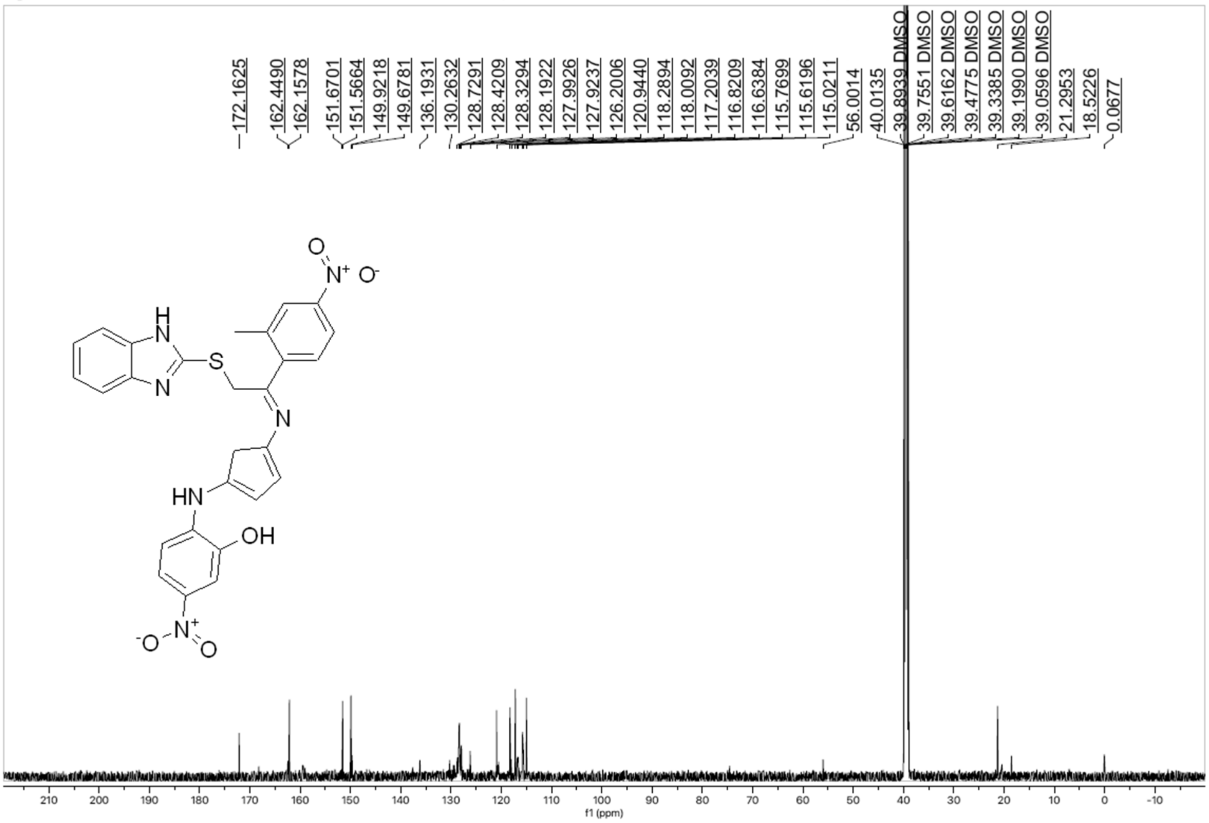

Figure S1. ^{13}CNMR for the compound **2** *(Z)-2-((4-((2-((1H-benzo[d]imidazol-2-yl)thio)-1-(2-methyl-4-nitrophenyl)ethylidene)amino)cyclopenta-1,3-dien-1-yl)amino)-5-nitrophenol* (2)

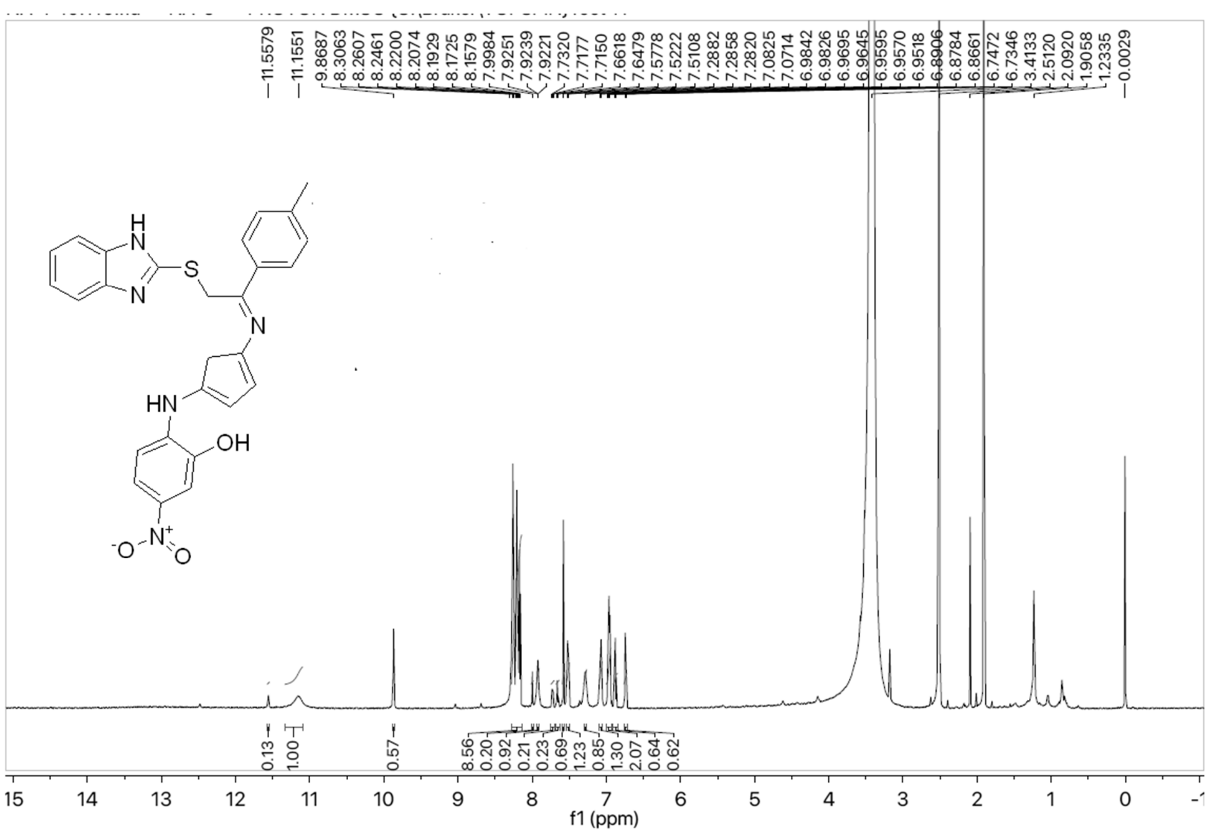

Figure S2. ^{1}HNMR for the compound **4** *(Z)-2-((4-((2-((1H-benzo[d]imidazol-2-yl)thio)-1-(p-tolyl)ethylidene)amino)cyclopenta-1,3-dien-1-yl)amino)-5-nitrophenol* (4).

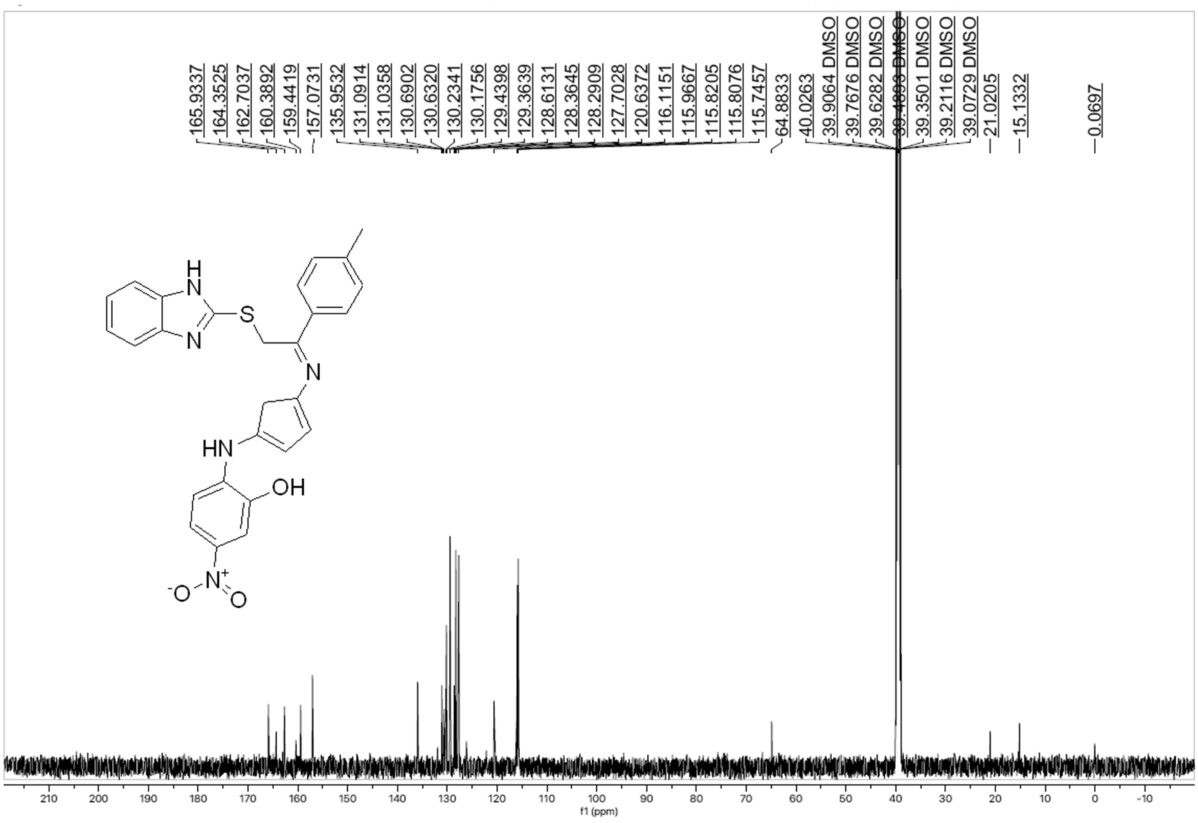

Figure S3. ^{13}CNMR for the compound **4** *(Z)-2-((4-((2-((1H-benzo[d]imidazol-2-yl)thio)-1-(p-tolyl)ethylidene)amino)cyclopenta-1,3-dien-1-yl)amino)-5-nitrophenol* (4)

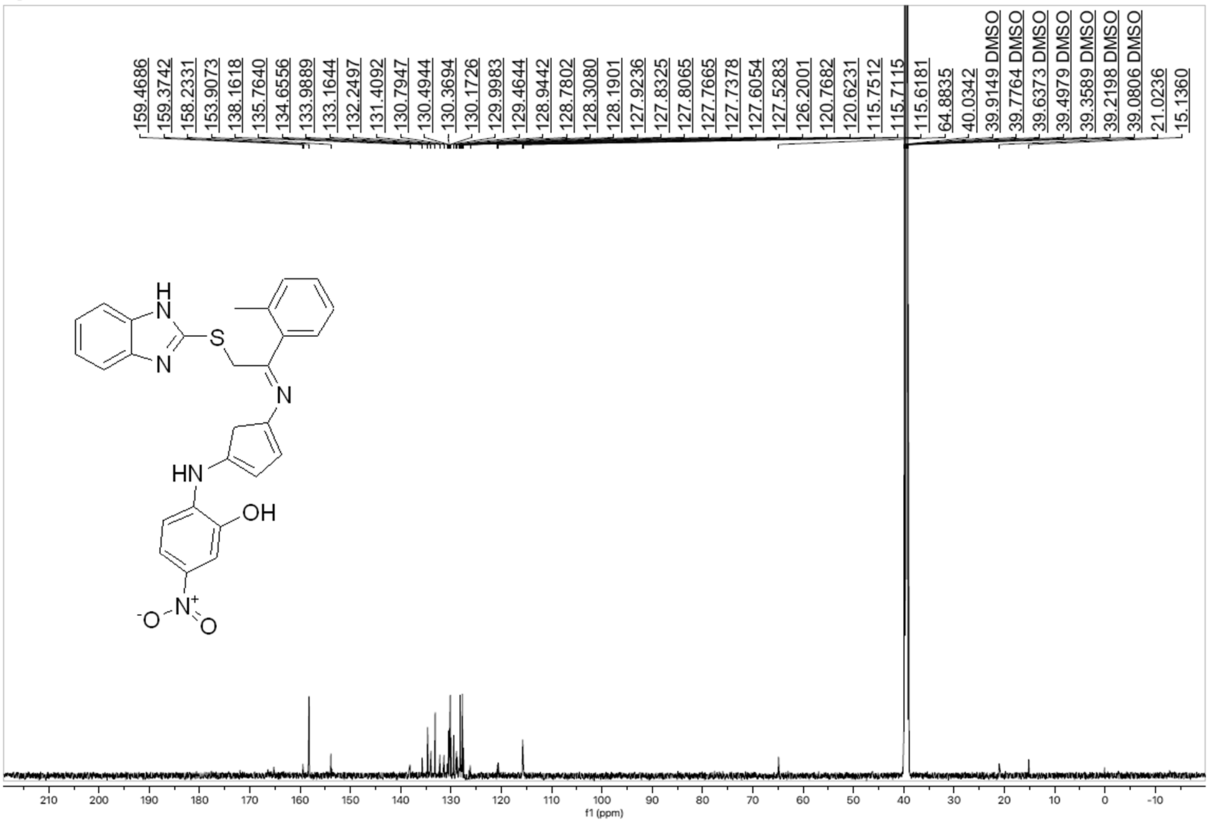

Figure S4. ^{13}CNMR for the compound **8** *(Z)-2-((4-((2-((1H-benzo[d]imidazol-2-yl)thio)-1-(o-tolyl)ethylidene)amino)cyclopenta-1,3-dien-1-yl)amino)-5-nitrophenol* (8)

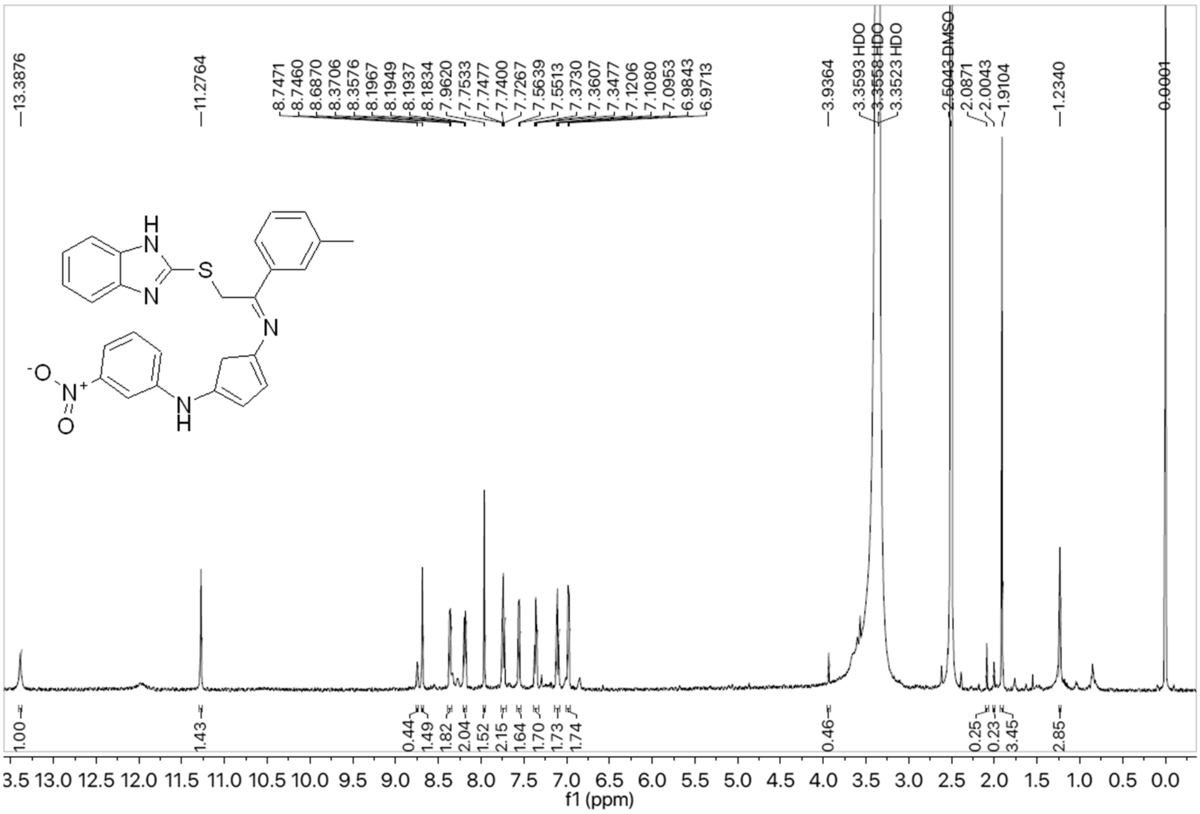

Figure S5. ^{1}HNMR for the compound **13** *(Z)-N-(4-((2-((1H-benzo[d]imidazol-2-yl)thio)-1-(m-tolyl)ethylidene)amino)cyclopenta-1,3-dien-1-yl)-3-nitroaniline* (13)

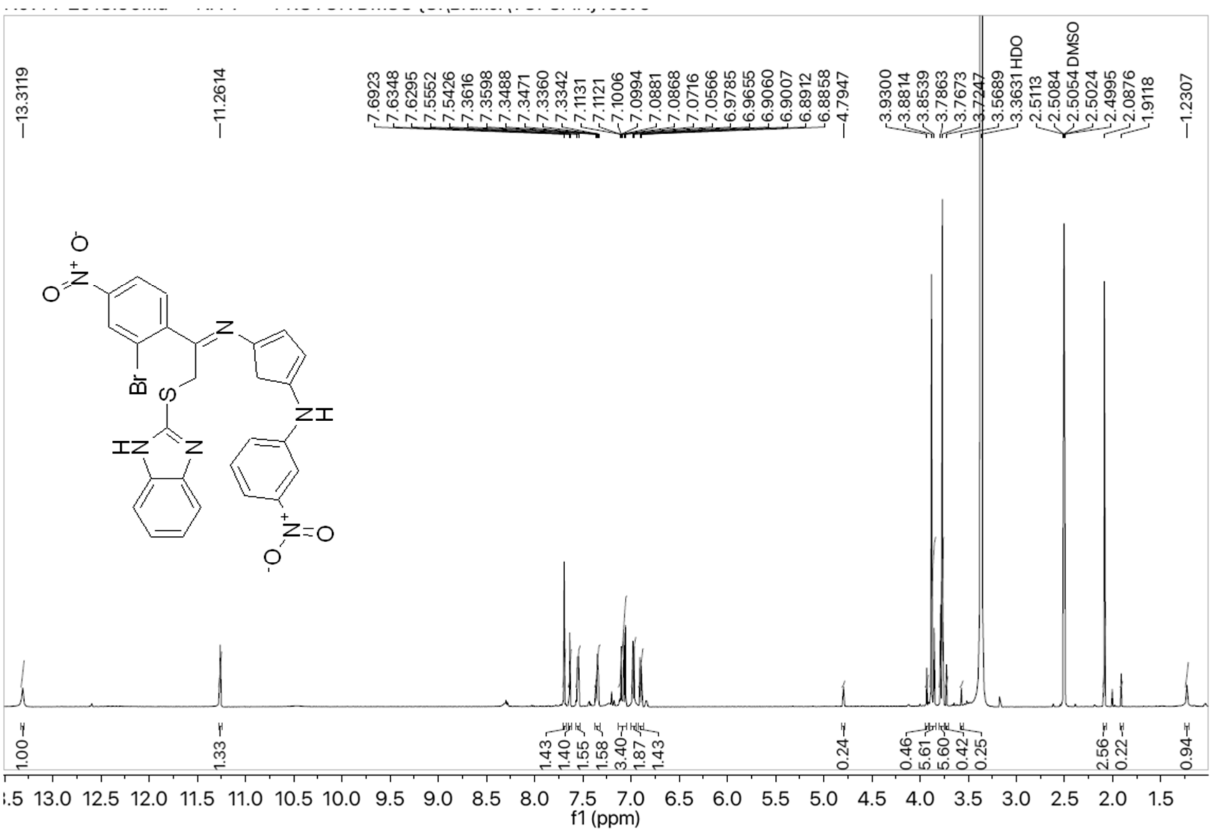

Figure S6. ^{1}HNMR for the compound **18** *(Z)-N-(4-((2-((1H-benzo[d]imidazol-2-yl)thio)-1-(2-bromo-4-nitrophenyl)ethylidene)amino)cyclopenta-1,3-dien-1-yl)-3-nitroaniline* (18)
